# Prospective evaluation of Gadoxetate-enhanced magnetic resonance imaging and computed tomography for hepatocellular carcinoma detection and transplant eligibility assessment with explant histopathology correlation

**DOI:** 10.1186/s40644-023-00532-3

**Published:** 2023-02-25

**Authors:** Kartik S. Jhaveri, Ali Babaei Jandaghi, Rajesh Bhayana, Khaled Y. Elbanna, Osvaldo Espin-Garcia, Sandra E. Fischer, Anand Ghanekar, Gonzalo Sapisochin

**Affiliations:** 1 Joint Department of Medical Imaging, University Health Network, Mount Sinai Hospital and Women’s College Hospital, University of Toronto, 610 University Ave, 3-957, Toronto, ON M5G 2M9 Canada; 2 Joint Department of Medical Imaging, University Health Network, Mount Sinai Hospital and Women’s College Hospital, Toronto, ON M5G 1X6 Canada; 3 Joint Department of Medical Imaging, University Health Network, Mount Sinai Hospital and Women’s College Hospital, University of Toronto, Toronto, ON M5G 2M9 Canada; 4 Department of Biostatistics, Princess Margaret Cancer Centre, University Health Network, Toronto, ON M5G 2C1 Canada; 5 Division of Biostatistics, Dalla Lana School of Public Health, University of Toronto, Toronto, Canada; 6 Department of Pathology, University Health Network and University of Toronto, Toronto, Ontario Canada; 7 University Health Network, Department of Surgery, Toronto General Hospital, University of Toronto, Toronto, ON M5G 2N2 Canada

**Keywords:** Carcinoma, Hepatocellular, Liver transplantation, Contrast media, Magnetic resonance imaging, Tomography, Computed, Gadoxetic acid, Milan criteria

## Abstract

**Background:**

We aimed to prospectively compare the diagnostic performance of gadoxetic acid-enhanced MRI (EOB-MRI) and contrast-enhanced Computed Tomography (CECT) for hepatocellular carcinoma (HCC) detection and liver transplant (LT) eligibility assessment in cirrhotic patients with explant histopathology correlation.

**Methods:**

In this prospective, single-institution ethics-approved study, 101 cirrhotic patients were enrolled consecutively from the pre-LT clinic with written informed consent. Patients underwent CECT and EOB-MRI alternately every 3 months until LT or study exclusion. Two blinded radiologists independently scored hepatic lesions on CECT and EOB-MRI utilizing the liver imaging reporting and data system (LI-RADS) version 2018. Liver explant histopathology was the reference standard. Pre-LT eligibility accuracies with EOB-MRI and CECT as per Milan criteria (MC) were assessed in reference to post-LT explant histopathology. Lesion-level and patient-level statistical analyses were performed.

**Results:**

Sixty patients (49 men; age 33–72 years) underwent LT successfully. One hundred four non-treated HCC and 42 viable HCC in previously treated HCC were identified at explant histopathology. For LR-4/5 category lesions, EOB-MRI had a higher pooled sensitivity (86.7% versus 75.3%, *p* <  0.001) but lower specificity (84.6% versus 100%, p <  0.001) compared to CECT. EOB-MRI had a sensitivity twice that of CECT (65.9% versus 32.2%, p <  0.001) when all HCC identified at explant histopathology were included in the analysis instead of imaging visible lesions only. Disregarding the hepatobiliary phase resulted in a significant drop in EOB-MRI performance (86.7 to 72.8%, p <  0.001). EOB-MRI had significantly lower pooled sensitivity and specificity versus CECT in the LR5 category with lesion size < 2 cm (50% versus 79%, *p* = 0.002 and 88.9% versus 100%, p = 0.002). EOB-MRI had higher sensitivity (84.8% versus 75%, *p* <  0.037) compared to CECT for detecting < 2 cm viable HCC in treated lesions. Accuracies of LT eligibility assessment were comparable between EOB-MRI (90–91.7%, *p* = 0.156) and CECT (90–95%, *p* = 0.158).

**Conclusion:**

EOB-MRI had superior sensitivity for HCC detection; however, with lower specificity compared to CECT in LR4/5 category lesions while it was inferior to CECT in the LR5 category under 2 cm. The accuracy for LT eligibility assessment based on MC was not significantly different between EOB-MRI and CECT.

**Trial registration:**

ClinicalTrials.gov Identifier: NCT03342677, Registered: November 17, 2017.

**Supplementary Information:**

The online version contains supplementary material available at 10.1186/s40644-023-00532-3.

## Key points

- Gadoxetic acid-enhanced Liver MRI had better diagnostic performance in some categories compared to Computed Tomography for the diagnosis of Hepatocellular carcinoma in patients with liver cirrhosis enlisted for transplantation.
- Gadoxetic acid-enhanced liver MRI had a significantly superior ‘real world’ or true sensitivity for hepatocellular carcinoma diagnosis versus computed tomography.
- Liver transplantation eligibility assessment was not significantly different between gadoxetic acid-enhanced liver MRI and computed tomography.

## Background

Liver transplantation (LT) is the treatment of choice for patients with hepatocellular carcinoma (HCC) confined to the liver who are not eligible for partial hepatic resection or ablation, while palliative therapies such as transarterial chemoembolization (TACE), radiotherapy, and systemic agents are recommended for those with more advanced disease [1]. According to EASL guidelines, LT is also recommended for patients with very early-stage HCC [2].

Preoperative imaging is relied upon not only for HCC detection but also for the determination of LT eligibility [3]. While different LT jurisdictions around the world employ slightly different criteria to determine LT eligibility, all rely upon diagnostic imaging studies to provide critical information about HCC size, number, and presence of macrovascular invasion to determine waitlist priority for LT candidates. For example, in the United States, LT candidates are required to have CECT or MRI every 3 months while awaiting transplantation to determine that their HCC burden falls within the Milan Criteria (MC); those who do not exceed MC receive additional MELD exception points until they undergo LT, while those who are found to exceed MC may be delisted [4]. Since awaiting LT can result in HCC progression, the Organ Procurement and Transplantation Network (OPTN)/United Network for Organ Sharing (UNOS) has developed a supplemental system for prioritization of patients with HCC meeting OPTN T2 criteria (a single HCC of 2–5 cm or three or fewer HCCs 1–3 cm) for LT and a downstaging protocol using locoregional therapies for those exceeding MC [5].

To date, limited studies have prospectively compared the diagnostic performance of CECT and MRI for detection of HCC in LT candidates [6–9], with no consensus on which of CECT, extracellular gadolinium-based contrast-enhanced MRI (EC-MRI), or MRI with hepatobiliary agents is superior [10]. CECT can be performed quickly and is more widely available compared to MRI but has lower contrast resolution with incremental radiation exposure [11]. Gadoxetate-enhanced Liver MRI (EOB-MRI) has had variable results compared to EC-MRI [12] and CECT depending on HCC size and diagnostic criteria utilized in LT candidates, with only a few studies utilizing liver explants as the reference standard [13, 14]. Notably, the lack of whole-liver explant correlation and retrospective evaluations have also probably overestimated imaging sensitivity for HCC diagnosis [15–17].

Thus, we aimed to prospectively compare the diagnostic performance of EOB-MRI and CECT for HCC detection and transplant eligibility in pre-LT cirrhotic patients with explant histopathology correlation.

## Methods

### Study participants

**Fig. 1:**
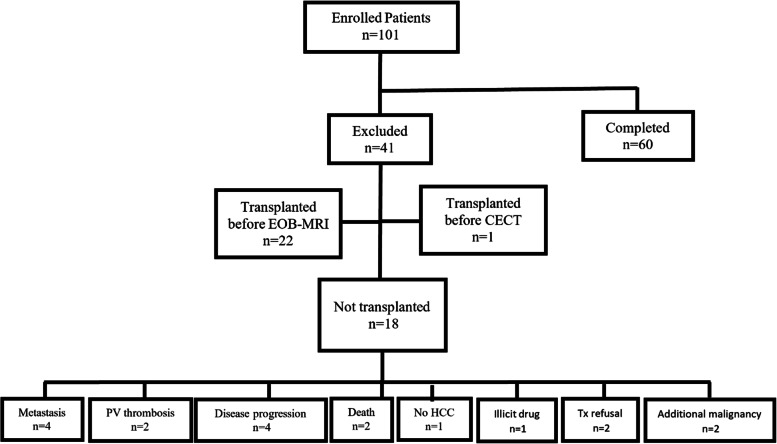
Patient enrollment flowchart. Abbreviations: EOB-MRI: Gadoxetic acid-enhanced MRI, CECT: Contrast-enhanced Computed Tomography, PV: portal vein, HCC: hepatocellular carcinoma, Tx: transplantation

This was a prospective, single-institution HIPAA compliant, and ethics-approved study. Between November 2017 and April 2021, written informed consent was obtained from consecutive patients following chart review in a pre-transplant clinic. Inclusion criteria were: (a) liver cirrhosis enlisted for LT with a high probability of undergoing transplantation within 12 months, and (b) diagnosed, suspected and or treated HCC with priority MELD points based on cancer diagnosis. The exclusion criteria were patient age < 18 years, low Glomerular filtration rate (GFR) (< 30 mL/min/1.73 m2), high bilirubin (> 3 mg/dl), pregnancy, MRI contraindications (pacemaker etc.), prior systemic HCC treatment, bridging therapies (TACE or ablation) between imaging and LT or removal from the LT waiting list. Patients underwent CECT and EOB-MRI alternately every 3 months until LT. Among 101 enrolled patients, the final cohort comprised 60 patients with exclusion of 41 patients (Fig. 1 and Table 1).

**Table 1:** Patient demographic and clinical characteristics

All patients	60
**Characteristic**	
**Mean age at surgery (range, years)**	62.5 ± 7.5 (33–72)
**Sex**	
Male	49 (81.7)
Female	11 (18.3)
**Cause of chronic liver disease**	
HCV	20 (33.3)
HBV	16 (26.7)
Alcohol	11 (18.3)
NASH	9 (15)
Other	4 (6.7)
**LRT (per patient)**	46 (76.7)
**Type of LRT (per lesion)**	
Total number of treated observations	134 (100)
RFA	78 (58.2)
TACE	15 (11.2)
TACE and RFA	29 (21.7)
BE	4 (3)
MWA	3 (2.2)
SBRT	1 (0.7)
Not specified	4 (3)
**Type of liver transplantation**	
Living donor	5 (8.3)
Deceased donor	55 (91.7)
**Time interval (day)**	Mean (SD), Median (range)
CT and EOB-MRI	90.3 (41.4), 84.5 (0.0–271.0)
CT and LT	100.7 (63.9), 101.5 (0.0–279.0)
EOB-MRI and LT	69.1 (49.7), 61 (3–192)
Last imaging and LT	39.8 (28.9), 35 (0–110)

Data in parentheses are numbers used to calculate the percentages

*BE* bland embolization, *LRT* local-regional therapy, *MWA* microwave ablation, *RFA* radiofrequency ablation, *SBRT* stereotactic body radiation therapy, *TACE* transarterial chemoembolization

### Imaging techniques

CECT was performed on either of two scanners (Aquilion ONE or Aquilion 64, Toshiba CA, USA;) with a standardized multiphasic liver protocol (Additional file 1). EOB-MRI was performed on a 1.5 T (Magnetom Avanto; Siemens Healthcare, Erlangen, Germany) or 3 T (Magnetom Verio with Tim system; Siemens Health care, Erlangen, Germany) MRI scanner with multichannel phased array coils (16 or 32 channels) using a standardized liver protocol (Additional file 2).

### Image evaluation

While patients were enrolled, and imaging scans were performed prospectively, EOB-MRI and CECT performed closest to LT were retrieved and de-identified for retrospective image evaluation from departmental PACS. Two abdominal radiologists independently reviewed CECT and EOB-MRI data with a gap of at least 4 weeks to minimize recall bias. They were blinded to the explant histopathology findings but knew that the patients were cirrhotic with or without prior interventional therapy and enlisted for LT.

#### Non-treated lesions

Using LI-RADS v2018, each reader assessed the presence or absence of major and ancillary imaging features for all non-treated hepatic lesions measuring ≥ 0.5 cm on EOB-MRI (Additional file 3) and CECT. Lesion characteristics recorded on EOB-MRI and CECT are summarized in Additional files 4 and 5. Subtraction images were reviewed to evaluate arterial phase hyperenhancement (APHE). Washout and capsule enhancement were determined on portal venous or additionally on equilibrium phase (CECT) [18]. The largest axial diameter was measured in portal venous or hepatobiliary phase and, if invisible on the sequence with best margin demarcation. Threshold growth was not included in the assessment as no prior imaging studies were included for analysis. LI-RADS score was assigned to each recorded lesion. Analyses were performed considering HCC diagnosis as an observation score of LR-4/5 and LR-5 alone.

#### Treated lesions

All treated lesions were evaluated based on the LI-RADS treatment response algorithm (TRA), and a LI-RADS treatment response (LR-TR) category was assigned as viable, nonviable, equivocal or nonevaluable (Additional file 6) [18].

### Reference standard

A liver pathologist with more than 15 years of experience examined all the liver explants. The explanted livers were routinely sectioned into 5-mm-thick axial slices, and all suspicious macroscopic, bulging, or discolored nodules at gross examination underwent histopathological evaluation. The pathology report included final diagnosis, tumor size and segment location, degree of tumor differentiation, presence of microvascular or macrovascular invasion, presence of capsule, degree of necrosis, and maximum size of viable tumor in treated observations. Tumor stage was reported according to the 8th edition staging system of the American Joint Committee on Cancer (AJCC) [19]. Complete pathologic necrosis (CPN) (100%) in treated observations was used as a reference standard for nonviable tumors, and non-CPN (< 100%) was used for viable HCC.

### Radiology-pathology correlation

A study investigator (ABJ) correlated the recorded observations on EOB-MRI and CECT with those on explant pathology reports based on lesion size and segment location. Lesions were matched if the difference between the pathologically and radiologically measured sizes was less than 10 mm, and no similar-sized lesion was observed in the same segment. Cholangiocarcinoma and mixed HCC-cholangiocarcinoma tumors were analyzed as non-HCC tumors. HCCs detected only on histopathology without corresponding LR-4 or 5 imaging observations in the same segment location were regarded as false-negative (FN), and the contrary was defined as false-positive (FP).

### Transplant allocation

Prospective LT allocation was determined as per Extended Toronto criteria (ETC), which offers LT irrespective of HCC size or number but requires no macrovascular invasion, extrahepatic disease, systemic cancer-related symptoms, or poorly differentiated tumors [20]. Thus, we evaluated simulated LT eligibility as per MC (single HCC ≤ 5 cm or 3 or fewer HCCs ≤3 cm, no vascular invasion and extrahepatic disease) [21] by each reader with EOB-MRI and CECT utilizing LI-RADS and OPTN criteria, verified in reference to explant histopathology. Regarding the OPTN criteria, all lesions detected on imaging were classified according to the OPTN classification system based on size and enhancement patterns (Additional file 7) [5]. In patients with treated observations (class 5T), the diameter of viable tumors was considered for determining LT eligibility. Therefore, patients having a single lesion (non-treated, class 5B; or treated, class 5T) with a maximum diameter of at least 2 cm and less than or equal to 5 cm, and those with up to 3 lesions (non-treated and/or treated), each greater than or equal to 1 cm and less than or equal to 3 cm (i.e., lesions in class 5A, class 5B only if less than 3 cm and class 5T only if greater than or equal to 1 cm and less than or equal to 3 cm) were considered eligible for LT.

### Statistical analysis

The diagnostic performance of EOB-MRI and CECT was compared for each reader based on a lesion-by-lesion level analysis of histopathologically confirmed HCC. Two lesion level analyses were performed: (i) wherein only the imaging detected lesions matched with corresponding histopathology correlation were included and (ii) wherein all lesions detected on histopathology irrespective of imaging visibility were included with the HCCs not detected on imaging being categorized as false negatives (FN). A *p*-value < 0.05 was considered statistically significant. Interobserver agreement was assessed via concordance rate (%) of diagnosis between the readers for EOB-MRI and CT. Reader-level scores were compared using McNemar tests. The sensitivity and corresponding 95% confidence intervals (CIs) of EOB-MRI and CECT in detecting HCC in correlation with the reference standard in pooled-reader analyses using a generalized estimating equations approach as previously described were calculated [22]. The performance of CECT and EOB-MRI on simulated LT allocation as per MC based on LI-RADS and OPTN criteria was also evaluated.

## Results

### Histopathologic results

One hundred seventeen non-treated liver lesions (mean size: 1.3 ± 0.65 cm, range: 0.5–3.5 cm) and 134 (mean size: 2.1 ± 1.32 cm, range: 0.1–6.0 cm) treated observations were recorded in 60 liver explants. Table 2 demonstrates the histopathologic findings of all non-treated lesions and treated observations. The distribution of non-treated HCCs and viable HCCs is shown in Additional file 8.

**Table 2:** Histopathologic characteristics of liver lesions at explant pathology

	***Non-treated lesions***				
	HCC	cHCC-CCA	CCA	Benign	Total
Number of lesions (%)	104 (88.9)	2 (1.7)	1 (0.9)	10 (8.5)	117 (100)
Mean size ± SD (range, cm)	1.3 ± 0.63 (0.5–3.5)	1.5 (0.5–2.5)	1.0 (NA)	1.3 ± 0.8 (0.6–32)	1.3 ± 0.65 (0.5–3.5)
Number in subgroups (%)					
0.5–0.9 cm	34 (29.1)	1 (0.9)	**–**	4 (3.4)	39 (33.3)
1–1.9 cm	56 (47.8)	**–**	1 (0.9)	4 (3.4)	61 (52.1)
≥ 2 cm	14 (12)	1 (0.9)	**–**	2 (1.7)	17 (14.6)
Differentiation (%)					
Well	9 (7.7)	**–**	**–**	**–**	9 (7.7)
Moderately	92 (78.6)	1 (0.9)	1 (0.9)	**–**	94 (80.3)
poorly	3 (2.6)	1 (0.9)	**–**	**–**	4 (3.4)
	***Treated observations***				
	Viable HCC	Viable cHCC-CCA		Non-viable HCC	Total
Number of lesions (%)	42 (31.3)	2 (1.5)		90 (67.2)	134 (100)
Whole mean size ± SD (range, cm)	2.9 ± 2.0 (0.6–10.5)	3.0 (2.0–4.0)		2.4 ± 1.3 (0.2–6.0)	2.6 ± 1.6 (0.2–10.5)
Viable mean size ± SD (range, cm)	1.6 ± 1.3 (0.1–5.5)	1.1 (0.5–1.6)		0 ± 0	1.5 ± 1.2 (0.1–5.5)
Number in subgroups (%)					
< 2 cm	32 (23.9)	2 (1.5)		36 (26.8)	70 (52.2)
≥ 2 cm	10 (7.5)	**–**		54 (40.3)	64 (47.8)
Differentiation states (%)					
Well	4 (3)	**–**		**–**	4 (3)
Moderately	36 (26.8)	**–**		**–**	36 (26.8)
poorly	2 (1.5)	2 (1.5)		**–**	4 (3)

*HCC* hepatocellular carcinoma, *cHCC-CCA* combined hepatocellular-cholangiocarcinoma, *CCA* cholangiocarcinoma, *SD* standard deviation

### Diagnostic performance of CECT and EOB-MRI for HCC detection

#### Observations with corresponding histopathologic abnormality (Table 3)

**Table 3:** Diagnostic performance of CECT and EOB-MRI observations with corresponding histopathologic abnormality

**LR-4 or 5 score as HCC**													
Size		**CECT**					**EOB-MRI**					*p*-value	
		Se	Sp	NPV	PPV	Acc	Se	Sp	NPV	PPV	Acc	Se	Sp
overall	R1	80.9 (38/47)	100 (4/4)	30.8 (4/13)	100 (38/38)	82.4 (42/51)	96.3 (78/81)	85.7 (6/7)	66.7 (6/9)	98.7 (78/79)	95.5 (84/88)		
	R2	69 (29/42)	100 (4/4)	23.5 (4/17)	100 (29/29)	71.7 (33/46)	76.6 (59/77)	83.3 (5/6)	21.7 (5/23)	98.3 (59/60)	77.1 (64/83)		
	Pooled	75.3	100				86.7	84.6				< 0.001	< 0.001
< 2 cm	R1	81 (34/42)	100 (2/2)	20 (2/10)	100 (34/34)	81.8 (36/44)	95.4 (62/65)	80 (4/5)	57.1 (4/7)	98.4 (62/63)	94.3 (66/70)		
	R2	64.9 (24/37)	100 (2/2)	13.3 (2/15)	100 (24/24)	66.7 26/39)	73.4 (47/64)	75 (3/4)	15 (3/20)	97.9 (47/48)	73.5 (50/68)		
	Pooled	73.4	100				84.5	77.8				1	1
≥ 2 cm	R1	80 (4/5)	100 (2/2)	66.7 (2/3)	100 (4/4)	85.7 (6/7)	100 (16/16)	100 (2/2)	100 (2/2)	100 (16/16)	100 (18/18)		
	R2	100 (5/5)	100 (2/2)	100 (2/2)	100 (5/5)	100 (7/7)	92.3 (12/13)	100 (2/2)	66.7 (2/3)	100 (12/12)	93.3 (14/15)		
	Pooled	90	100				96.6	100				0.452	0.452
**LR-5 score as HCC**													
size		**CECT**					**EOB-MRI**					*p*-value	
		Se	Sp	NPV	PPV	Acc	Se	Sp	NPV	PPV	Acc	Se	Sp
overall	R1	86.5 (32/37)	100 (4/4)	44.4 (4/9)	100 (32/32)	87.8 (36/41)	54.2 (32/59)	100 (7/7)	20.6 (7/34)	100 (32/32)	59.1 (39/66)		
	R2	68.6 (24/35)	100 (4/4)	26.7 (4/15)	100 (24/24)	71.8 (28/39)	50 (30/30)	83.3 (5/6)	14.3 (5/35)	96.8 (30/31)	53 (35/66)		
	Pooled	77.8	100				52.1	92.3				0.243	0.243
1–1.9 cm	R1	87.5 (28/32)	100 (2/2)	33.3 (2/6)	100 (28/28)	88.2 (30/34)	48.8 (21/43)	100 (5/5)	18.5 (5/27)	100 (21/21)	54.2 (26/48)		
	R2	70 (21/30)	100 (2/2)	18.2 (2/11)	100 (21/21)	71.9 (23/32)	51.1 (24/47)	75 (3/4)	11.5 (3/26)	96 (24/25)	52.9 (27/51)		
	Pooled	79	100				50	88.9				0.002	0.002
≥ 2 cm	R1	80 (4/5)	100 (2/2)	66.7 2/3)	100 (4/4)	85.7 (6/7)	68.8 (11/16)	100 2/2)	28.6 (2/7)	100 (11/11)	72.2 (13/18)		
	R2	60 (3/5)	100 (2/2)	50 (2/4)	100 (3/3)	71.4 (5/7)	46.2 (6/13)	100 (2/2)	22.2 (2/9)	100 (6/6)	53.3 8/15)		
	Pooled	70	100				58.6	100				1	1

Data are percentages, with numerators and denominators in parentheses

*Acc* accuracy, *CECT* contrast-enhanced computed tomography, *EOB-MRI* Gadoxetic acid-enhanced MRI, *NPV* negative predictive value, *PPV* positive predictive value, *R1* reader 1, *R2* reader 2, *Se* sensitivity, *Sp* specificity

***Non-treated lesions***

*LR-4/5 score as HCC*

**Fig. 2:**
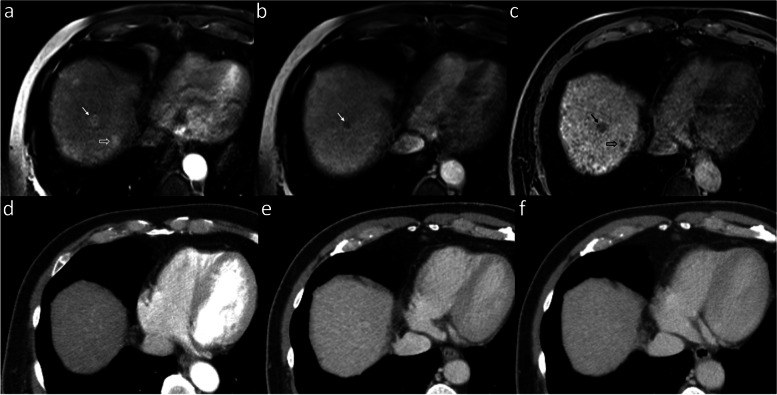
Superiority of EOB-MRI compared to CECT in detecting HCC. In a 68-year-old male, EOB-MRI shows a 10-mm arterial phase hyperenhancing HCC (arrow, **a**) in segment 8 with nonperipheral washout (arrow, **b**) and obvious hypointensity on HBP (arrow, **c**) in keeping with LR5. However, the lesion was invisible on all phases of CECT (**d**-**f**). An additional 8-mm arterial hyperenhancing lesion is seen adjacent to the inferior vena cava (hollow arrow, **a**) without venous washout. Considering HBP hypointensity (hollow arrow, **c**), it was categorized as LR-4. Again, the lesion was invisible on CECT. Histopathologic examination of liver explant confirmed HCC at both these locations. Abbreviations: CECT: contrast-enhanced computed tomography, EOB-MRI: Gadoxetate-enhanced-MRI, HCC: hepatocellular carcinoma, HBP: hepatobiliary phase

Without size consideration, HCC detection sensitivity and accuracy were higher for both readers with EOB-MRI versus CECT. The pooled sensitivity was significantly greater with EOB-MRI (Fig. 2), while the pooled specificity was significantly lower with EOB-MRI versus CECT. For lesions < 2 cm, EOB-MRI had higher sensitivity and accuracy for both readers and only for one reader in the ≥2 cm group. EOB-MRI had higher pooled sensitivities for both < 2 cm and ≥ 2 cm lesions and a lower pooled specificity for the < 2 cm group, although without reaching statistical significance.

*LR-5 score as HCC*

Regardless of size, the sensitivity and accuracy were higher with CECT compared to EOB-MRI for both readers. However, pooled sensitivity with CECT was not-significantly higher than EOB-MRI. The pooled specificity of CECT was again not-significantly greater than EOB-MRI. For HCC size of 1–1.9 cm, a statistically significant lower pooled sensitivity was observed for EOB-MRI vs CECT with a statistically significant higher pooled specificity with CECT. For HCC ≥ 2 cm, the pooled sensitivities were not significantly different, and specificities were equivalent.

*LR-3 score*

All LR-3 lesions scored by both readers on EOB-MRI and CECT proved to be HCCs at explant histopathology, hence were considered false negative in our analysis. Reader 1 scored fewer LR-3 lesions on EOB-MRI versus CECT (2 vs 8, respectively), with similar occurrences for reader 2 (11 vs 9, respectively). The final diagnosis of LR-2 and LR-3 lesions is summarized in Additional file 9.

*Added value of HBP*

**Fig. 3:**
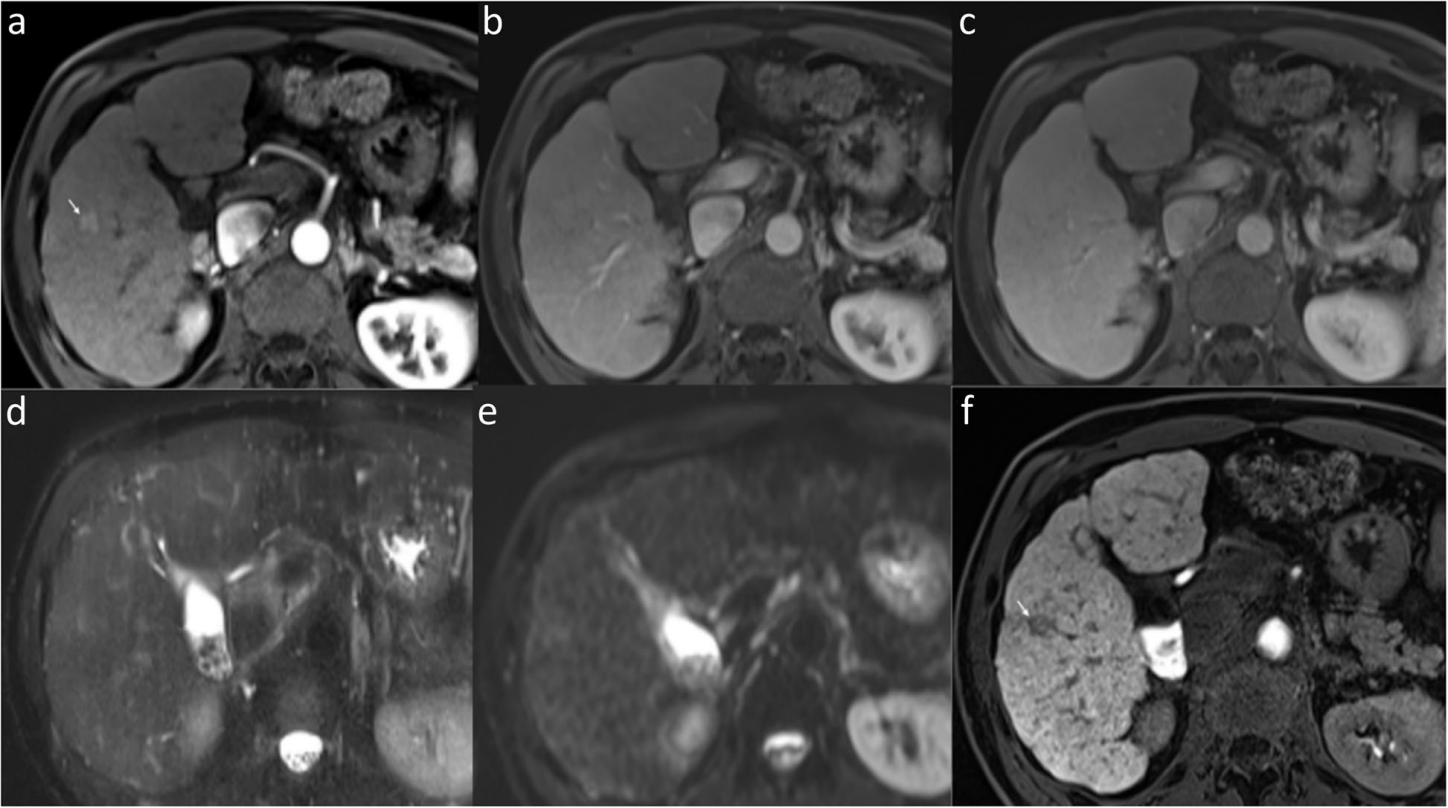
Impact of HBP on LI-RADS scoring. EOB-MRI in a 53-year-old male shows a 14-mm arterial phase hyperenhancing lesion (arrow, **a**) within segment 5 without washout (**b** and **c**) and without T2 hyperintense signal (**d**) or diffusion restriction (**e**) but with distinct HBP hypointensity (**f**). The LI-RADS score was upgraded to LR-4 based on the HBP signal as an ancillary feature. The lesion was confirmed as HCC at explant histopathology. Abbreviations: CECT: contrast-enhanced computed tomography, EOB-MRI: Gadoxetate-enhanced-MRI, HCC: hepatocellular carcinoma, HBP: hepatobiliary phase, LI-RADS: Liver Imaging Reporting and Data System

Irrespective of size, rescoring of observations disregarding HBP as an ancillary LI-RADS feature resulted in a significant drop in pooled sensitivity of LR-4/5 as HCC (86.7 to 72.8%, *p* <  0.001), whereas the specificity remained unchanged (84.6%). The disregard of HBP signal specifically impacted the HCC <  2 cm with a significant drop in pooled sensitivity (84.5 to 69.0%, *p* < 0.001) while specificity (77.8%) remained unchanged (Fig. 3).

***Treated observations***

**Fig. 4:**
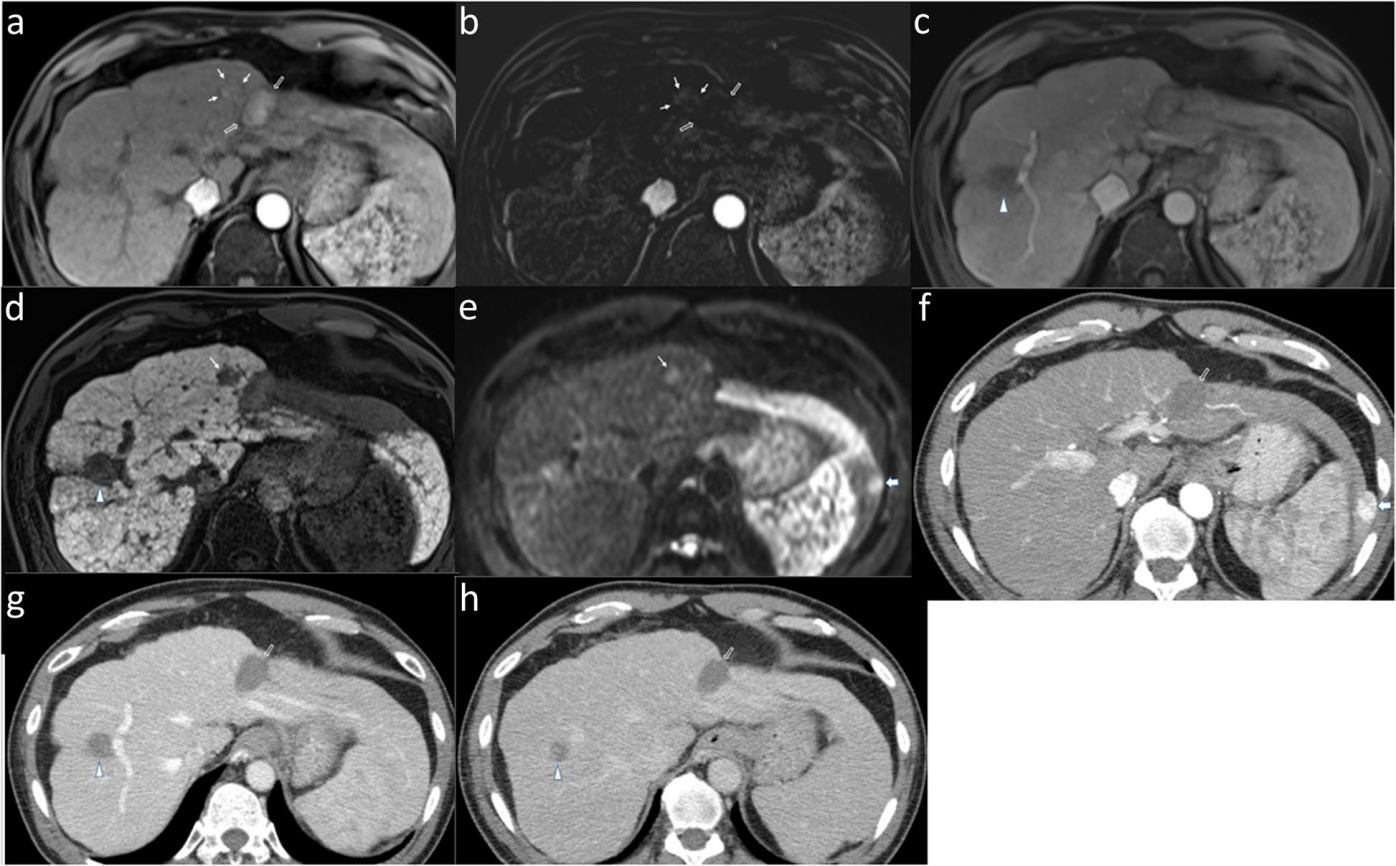
Superior diagnostic performance of EOB-MRI in detecting < 2 cm-sized viable HCC (LR-TR viable) over CECT. In a 53-year-old man with a history of radiofrequency ablation in segment 3, EOB-MRI (arrows, **a**) demonstrates a 17-mm nodular enhancement which is more conspicuous on the arterial subtraction image (arrows, **b**), anterolateral to the treated observation (hollow arrows, **a** and **b**), without venous phase washout (**c**), but with HBP hypointensity (arrow, **d**) and diffusion restriction (thin arrow, **e**). The treated observation was assigned a viable HCC category on EOB-MRI. On CECT (**f**–**h**), the treated observation (hollow arrow, **f**-**h**) was assigned as non-viable due to lack of nodular arterial phase enhancement. A well-differentiated HCC was detected along the treated observation on histopathologic examination of the liver explant. Additionally demonstrated is a non-enhancing 23-mm treated observation (LR-TR non-viable) in segment 7/8 on EOB-MRI and CECT (arrowheads) with proven complete necrosis on histopathology. As a variant of hepatic morphology, the elongated left liver lobe extends laterally to surround the spleen (beaver tail liver), harboring an 18-mm histopathological proven non-treated HCC (thick arrow, **f**). This lesion manifested typical findings of HCC, including arterial hyperenhancement and washout (LR-5) on both EOB-MRI (not shown) and CECT. Image 5e (thick arrow) depicts the lesion manifesting diffusion restriction. Abbreviations: CECT: contrast-enhanced computed tomography, EOB-MRI: Gadoxetate-enhanced-MRI, HCC: hepatocellular carcinoma, HBP: hepatobiliary phase, LR-TR: Liver Imaging Reporting and Data System-Treatment response

Regardless of size, the overall sensitivity, specificity, and accuracy for detection of viable HCC were comparable between readers with EOB-MRI and CECT, with no statistically significant differences in pooled sensitivity and specificity (Table 4). The pooled sensitivity and specificity for detecting viable HCC <  2 cm were significantly greater with EOB-MRI versus CECT (Fig. 4). Further, the pooled sensitivity and specificity for viable HCC ≥ 2 cm were also higher with EOB-MRI versus CECT; however, the differences were statistically insignificant.

**Table 4:** Diagnostic performance of CECT and EOB-MRI for detecting viable HCC with corresponding histopathologic abnormality

Size		CECT					EOB-MRI					*P*-value	
		Se	Sp	NPV	PPV	Acc	Se	Sp	NPV	PPV	Acc	Sen	Sp
overall	R1	58.3 (21/36)	86.6 (58/67)	79.5 (58/73)	70 (21/30)	76.7 (79/103)	66.7 (22/33)	88.5 (54/61)	83.1 (54/65)	75.9 (22/29)	80.9 (76/94)		
	R2	54.5 (18/33)	91.7 (66/72)	81.5 (66/81)	75 (18/24)	80 (84/105)	60 (21/35)	95.8 (69/72)	83.1 (69/83)	87.5 (21/24)	84.1 (90/107)		
	Pooled	56.5	89.2				63.2	92.5				0.312	0.312
< 2 cm	R1	81 (17/21)	50 (9/18)	69.2 (9/13)	65.4 (17/26)	66.7 (26/39)	87.5 (14/16)	50 (7/14)	77.8 (7/9)	66.7 (14/21)	70 (21/30)		
	R2	66.7 (10/15)	75 (12/16)	70.6 12/17)	71.4 10/14)	71 (22/31)	82.4 (14/17)	83.3 (10/12)	76.9 (10/13)	87.5 (14/16)	82.8 (24/29)		
	Pooled	75	61.8				84.8	65.4				0.037	0.037
≥ 2 cm	R1	26.7 (4/15)	100 (49/49)	81.7 (49/60)	100 (4/4)	82.8 (53/64)	47.1 (8/17)	100 (45/45)	83.3 (45/54)	100 (8/8)	85.5 (53/62)		
	R2	44.4 (8/18)	96.4 (54/56)	84.4 54/64)	80 8/10)	83.8 (62/74)	38.9 (7/18)	98.3 (59/60)	84.3 (59/70)	87.5 (7/8)	84.6 (66/78)		
	Pooled	36.4	98.1				42.9	99				0.504	0.504

Data are percentages, with numerators and denominators in parentheses

*Acc* accuracy, *CECT* contrast-enhanced computed tomography, *EOB-MRI* Gadoxetic acid-enhanced MRI, *NPV* negative predictive value, *PPV* positive predictive value, *R1* reader 1, *R2* reader 2, *Se* sensitivity, *Sp* specificity

#### ‘Real-world’ (true) sensitivity considering all histopathological proven HCC

***Non-treated lesions (***Table 5***)***

The ‘real-world’ or true sensitivity for HCC detection as per LR-4/5 score was significantly superior with EOB-MRI versus CECT for both readers, regardless of HCC size, and the pooled sensitivity of EOB-MRI (65.9%) was more than twice that of CECT (32.2%).

**Table 5:** Real-world sensitivity of LR-4/5 and viable LR-TR for HCC detection with CECT versus EOB-MRI

**LR-4/5 (all HCC ≥ 5 mm at histopathology)**				
Size		**CECT**	**EOB-MRI**	*p*-value
Overall	R1	36.5 (38/104)	75 (78/104)	< 0.001
	R2	27.9 (29/104)	56.7 (59/104)	< 0.001
	pooled	32.2 (67/208)	65.9 (137/208)	< 0.001
**Treated observations (all viable HCC at histopathology)**				
Size		**CECT**	**EOB-MRI**	*p*-value
Overall	R1	47.6 (20/42)	50 (21/42)	1.00
	R2	42.9 (18/42)	50 (21/42)	0.66
	pooled	45.2 (38/84)	50 (42/84)	0.64

Data are percentages, with numerators and denominators in parentheses

*CECT* contrast-enhanced computed tomography, *EOB-MRI* Gadoxetic acid-enhanced MRI, *R1* reader 1, *R2* reader 2

***Treated observations***

Forty-two viable HCCs were identified in 134 treated observations on histopathology. Regardless of size, EOB-MRI had a marginally higher true sensitivity versus CECT utilizing LI-RADS TRA; however, the differences were statistically insignificant.

### Simulated LT eligibility

**Fig. 5:**
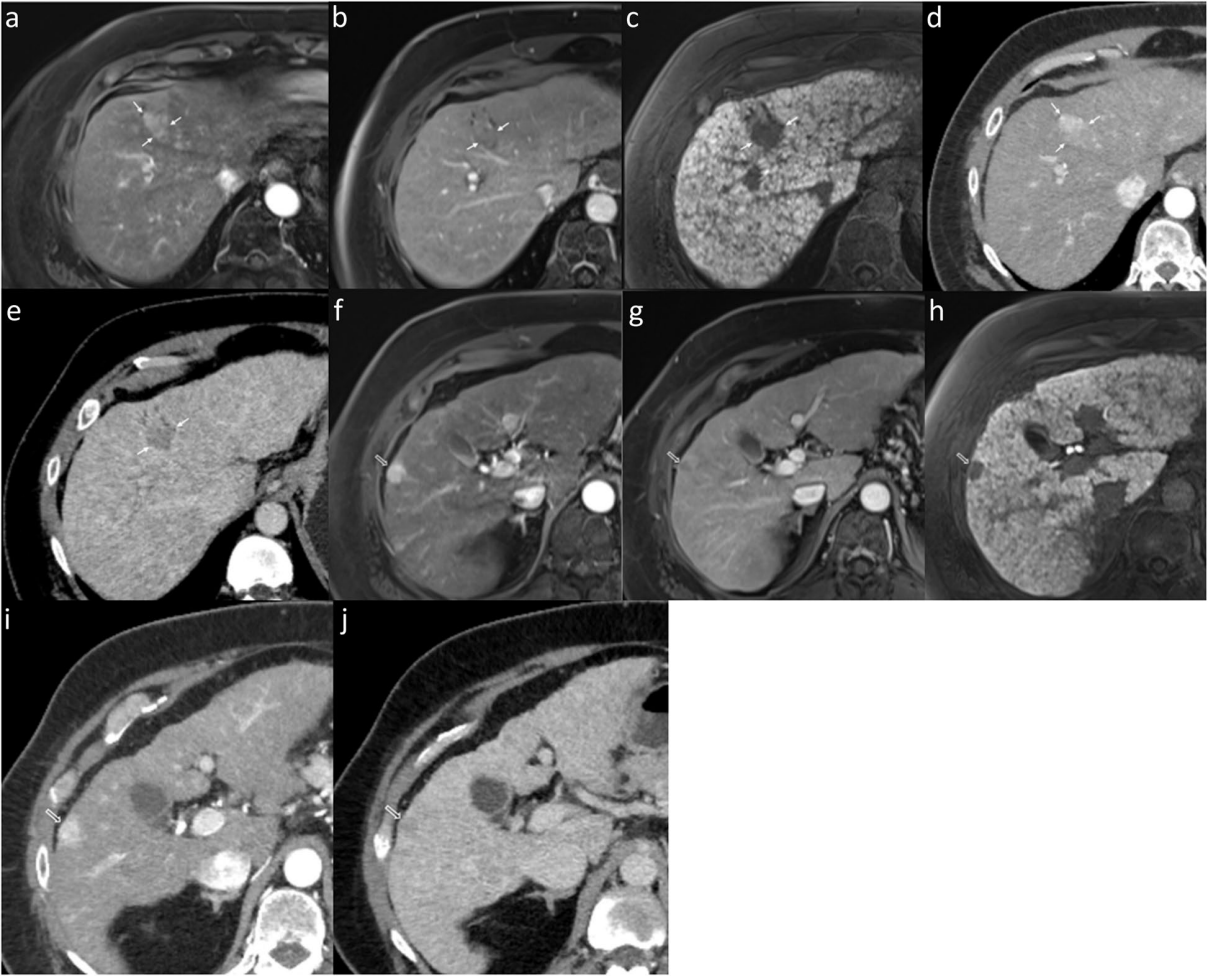
Discordance in LT eligibility as per MC from HCC diagnosis by OPTN criteria and LI-RADS. In a 62-year-old man, EOB-MRI (**a**–**c**) and CECT (**d** and **e**) demonstrated a 28-mm observation with non-rim APHE (arrows, **d**) and washout (arrows, **b** and **e**), but without enhancing capsule in segment 4a/8. The lesion was categorized/classified as LR-5 and 5B by LI-RADS and OPTN criteria, respectively. Additionally, there were four 1–1.9 cm-sized LR-5 observations (one is shown in segment 5; hollow arrow, **f**–**j**), with non-rim APHE (**f** and **i**) and washout (**g** and **j**) but no enhancing capsule. Lesions demonstrated HBP hypointensity (arrows, **c**; hollow arrow, **h**). None of these five observations were eligible to be classified as 5A according to OPTN criteria due to lack of delayed peripheral enhancement, and therefore, based on the presence of a single 5B observation, the patient would be deemed as meeting the MC. In contrast, he would be considered beyond the MC according to LI-RADS categories (presence of five LR-5 observations). Since histopathologic examination of the liver explant revealed five HCC, the patient was indeed beyond MC and unsuitable for liver transplantation. Abbreviations: APHE: arterial phase enhancement, CECT: contrast-enhanced computed tomography, EOB-MRI: Gadoxetate-enhanced-MRI, HCC: hepatocellular carcinoma, HBP: hepatobiliary phase, LI-RADS: Liver Imaging Reporting and Data System, MC: Milan criteria, OPTN: Organ Procurement and Transplantation Network

According to explant histopathology findings, 88.3% (53/60) of patients would have been eligible for LT based on MC (Table 6). The overall accuracy in determining LT eligibility using EOB-MRI and CECT was comparable between LI-RADS and OPTN criteria for both readers, and the differences were statistically insignificant (EOB-MRI: *p* = 0.156; CT: *p* = 0.158). However, both readers showed lower accuracies with CECT and EOB-MRI in detection of patients exceeding versus those meeting MC in reference to histopathology (LI-RADS: *p* = 0.004; UNOS guidelines: *p* = 0.03). Both readers obtained higher accuracy (with both CECT and EOB-MRI) in predicting patients exceeding MC with HCC diagnosis as per LI-RADS scoring compared to OPTN criteria (*p* = 0.06) (Fig. 5).

**Table 6:** Accuracy of CECT and EOB-MRI for LT eligibility as per Milan criteria with histopathological correlation

	**LI-RADS**			
	Reader 1		Reader 2	
	CECT	EOB-MRI	CECT	EOB-MRI
Meeting MC	92.5 (49/53)	94.3 (50/53)	98.1 (52/53)	94.3 (50/53)
Exceeding MC	85.7 (6/7)	71.4 (5/7)	71.4 (5/7)	71.4 (5/7)
Overall Accuracy	91.7 (55/60)	91.7 (55/60)	95 (57/60)	91.7 (55/60)
	**OPTN criteria**			
	Reader 1		Reader 2	
	CECT	EOB-MRI	CECT	EOB-MRI
Meeting MC	96.2 (51/53)	94.3 (50/53)	98.1 (52/53)	98.1 (52/53)
Exceeding MC	42.9 (3/7)	57.1 (4/7)	42.9 (3/7)	42.9 (3/7)
Overall Accuracy	90 (54/60)	90 (54/60)	91.7 (55/60)	91.7 (55/60)

Data are percentages, with numerators and denominators in parentheses

*CECT* contrast-enhanced computed tomography, *EOB-MRI* Gadoxetic acid-enhanced MRI, *LI-RADS* Liver Imaging Reporting and Data System, *MC* Milan criteria, *OPTN* Organ Procurement and Transplantation Network

### Interobserver agreement

Interobserver agreement was substantial for EOB-MRI (κ = 0.746, CI 0.635–0.858] and CECT (κ = 0.712; CI: 0.541–0.883) for all observations with a corresponding histopathologic abnormality. CECT had greater interobserver agreement than EOB-MRI for categorizing LR-4/5 (κ = 0.572; CI: 0.219–0.926 vs κ = 0.406; CI: 0.053–0.759). For LR-TR categorization, the interobserver agreement was substantial for EOB-MRI (κ = 0.751, CI: 0.585–0.916) and CECT (κ = 0.708, CI: 0.457–0.958). Interobserver agreement for determination of LT eligibility was moderate for LI-RADS (κ = 0.496 [95% CI, 0.239–0.754] and OTPN criteria (κ = 0.565 [95% CI, 0.209–0.922]).

## Discussion

Our study demonstrated that EOB-MRI had an overall superior sensitivity but lower specificity compared to CECT for HCC diagnosis in non-treated LR-4/5 lesions referenced against explant histopathology. EOB-MRI was superior to CECT on analysis by size (< 2 cm versus > 2 cm), although without statistical significance. In the LR-5 category, although EOB-MRI and CECT had statistically similar pooled diagnostic performance without consideration of HCC size, EOB-MRI was inferior to CECT in the < 2 cm subgroup. HCC detection sensitivity and specificity with CECT and EOB-MRI have wide reported variability in the LR-4/5 and LR-5 categories defined by LI-RADSv2018 [12, 23–25]. A prior meta-analysis of 27 studies concluded that since EOB-MRI showed significantly higher sensitivity and diagnostic accuracy without substantial loss of specificity versus CECT, it should be the preferred imaging modality for small HCC ≤ 2 cm. However, pooling of predominantly retrospective data with heterogeneous inclusion criteria and reference standards may have resulted in overestimated diagnostic performance [17]. Only one retrospective study has utilized liver explants as the sole reference standard comparing CECT versus EOB-MRI for HCC detection based on prior LI-RADS v2017 criteria [13]. They reported superiority of MRI over CECT, without significant differences between EC-MRI and EOB-MRI, particularly for lesions measuring 1–1.9 cm, although EOB-MRI outperformed EC-MRI for per-patient HCC detection.

Evaluation of whole-liver explant parenchyma has the merit of detecting HCC invisible on imaging, thereby getting truer imaging sensitivity estimates. The ‘real world’ or true sensitivities of EOB-MRI and CECT in LR-4/5 categories were lower in our study when analyses included all HCC diagnosed at explant histopathology irrespective of a corresponding imaging observation. A prior meta-analysis and a recent study have reported lower sensitivities of EOB-MRI in studies wherein liver explant was the only reference standard [15, 26] in line with our comparable lower real-world sensitivities with EOB-MRI. Nevertheless, in a ‘real-world’ scenario, EOB-MRI was significantly superior to CECT in the LR-4/5 category.

HBP signal as an AF for adjusting LI-RADS category significantly improved EOB-MRI sensitivity in LR-4/5 observations in this study, regardless of size and for < 2 cm group, without significant impact on specificity. A prior retrospective study reported no significant differences between CECT and EOB-MRI for HCC diagnosis with LR-4/5 and LR-5 categories [23]. However, AFs were only utilized to assign LR-1 or 2 categories which could have lowered EOB-MRI results. In the LR-5 category, we found an overall insignificantly higher sensitivity for CECT over EOB-MRI with a similar PPV of 100% on both imaging modalities, in alignment with their results. Although LI-RADSv2018 can be utilized for interpretation with hepatobiliary contrast agents (HBAs), unlike with extracellular contrast agents, only portal venous phase hypointensity qualifies as the washout appearance. This has implications for HCC diagnosis in the LR-5 category [26–28], probably contributing to lowered diagnostic performance of EOB-MRI in the < 2 cm HCC subgroup in our study, like few previous studies [29–31]. Although HBP hypointensity is not intended to upgrade to LR-5, it can improve HCC detection sensitivity without impairing specificity with EOB-MRI [32], as depicted by our results in the LR-4/5 category.

In our study, EOB-MRI was superior to CECT for detection of < 2 cm viable HCC in treated lesions as per LI-RADS TRA. However, without size consideration, there were no significant differences between the two modalities like in some prior studies [33, 34]. A prior study by Bae et al. [35] reported higher sensitivity of HBA-enhanced MRI over CECT. This discrepancy could be attributable to a greater percentage of conventional TACE–treated lesions in their study (73.4%) impacting accurate assessment of APHE with CECT [36]. Arterial phase subtraction images, through better visualization of APHE, have been reported to improve the sensitivity of EOB-MRI in detection of viable HCC after LRT [37] and is routinely performed in all EOB-MRI at our institution.

In this study, EOB-MRI and CECT had equivalent performances for assessing simulated LT eligibility based on MC with LI-RADSv2018 and OPTN criteria in reference to explant histopathology, even as lower accuracies were observed in prediction of patients exceeding MC. Although recent studies have demonstrated moderate to high accuracy for determining LT eligibility based on MC using CECT and EOB-MRI with LI-RADSv2018 and OPTN, however in these retrospective studies [25, 38], patients with LRT for HCC before LT were excluded. Since both non-treated and viable HCC in treated observations require to be considered for MC before LT, our study results convey more real-world accuracies. We observed a higher accuracy for identifying unsuitable LT candidates based on MC with LI-RADS versus OPTN, although statistically insignificant. Contrary to our results, a lower accuracy of LI-RADSv2018 (57.9%) vs OPTN (85.7%) has been reported in this scenario previously [38]. This difference could again be due to the exclusion of patients with treated lesions from that study. Even though classic HCC features and threshold growth criteria for definite HCC by LI-RADS (LR-5) and OPTN (OPTN-5) are similar in both systems, there are some important differences [5, 18, 39]. While OPTN criteria do not incorporate HBP features, our results support using EOB-MRI for OPTN classification, given the high accuracy obtained for LT eligibility.

We must acknowledge some limitations of this study. This was a single-center study with a limited patient cohort; however, all patients were enrolled prospectively, and explant histopathology correlation was available for the entire study cohort. The lack of using threshold growth in this study for assignment of LI-RADS categories may have underestimated the sensitivity for HCC diagnosis but is applicable to both modalities. The mean time interval between CECT and LT was 1 month longer than the time between EOB-MRI and LT, which could have led to changes in HCC characteristics, thereby impacting imaging evaluation variably between CECT and EOB-MRI. We acknowledge that the three-month interval may change tumor size for aggressive HCC, and it would be ideal to have both CECT and EOB-MRI performed closely before LT. However, since we could not predict the exact timepoint of LT, it was not feasible to perform both EOB-MRI and CECT at every 3-month follow-up interval. It can be challenging to precisely match every lesion recorded on imaging to the liver explant, particularly with small lesion size and because of the shrinkage effects of formalin fixation. Lastly, since we used CPN or non-CPN at histopathologic examination as the reference standard for viable HCC in treated lesions, microscopically viable HCC could have led to underestimation of LR-TR viable category.

## Conclusions

EOB-MRI has superior diagnostic performance for HCC diagnosis in pre-LT cirrhotic patients over CECT in specific categories, while it was inferior to CECT for LR-5 observations less than 2 cm. While both EOB-MRI and CECT have a lower diagnostic performance in a real-world scenario, EOB-MRI outperforms CECT therein. At a patient level, LT eligibility assessment appears to be comparable between EOB-MRI and CECT; however, both modalities have relatively lower accuracies for identifying patients exceeding MC compared to those within MC.

## Supplementary Information

**Additional file 1.** Multiphasic Liver CT protocol (Aquilion 64).

**Additional file 2.** MRI Protocol for Gadoxetic acid-enhanced liver MRI (EOB-MRI).

**Additional file 3.** LI-RADS v2018 – Major and Ancillary Features with EOB-MRI.

**Additional file 4.** Lesion characteristics evaluated on EOB-MRI.

**Additional file 5.** Lesion characteristics evaluated on Contrast-enhanced CT scan.

**Additional file 6.** CECT and EOB-MRI treatment response categories.

**Additional file 7.** OPTN classification system for lesions seen on imaging of cirrhotic livers.

**Additional file 8.** Distribution of non-treated hepatocellular carcinoma (HCC) and viable HCC in explants.

**Additional file 9.** Histopathology results for LR-2 and LR-3 lesions scored by readers on EOB-MRI and CECT.

## Data Availability

The datasets used and/or analysed during the current study are available from the corresponding author on reasonable request.

## Abbreviations

APHE: Arterial phase hyperenhancement
CPN: Complete pathologic necrosis
CECT: Contrast-enhanced Computed tomography
DWI: Diffusion-weighted imaging
EC-MRI: Extracellular gadolinium-based contrast-enhanced MRI
EOB-MRI: Gadoxetic acid-enhanced MRI
ETC: Extended Toronto criteria
FP: False positive
HCC: Hepatocellular carcinoma
HBP: Hepatobiliary phase
LI-RADS: Liver imaging reporting and data system
LI-RADS: Treatment response algorithm (LI-RADS TRA)
LT: Liver transplantation
MC: Milan criteria
MELD: Model for end-stage liver disease
NPV: Negative predictive value
OPTN: Organ Procurement and Transplantation Network
PPV: Positive predictive value
TACE: Transarterial chemoembolization
UNOS: United network for organ sharing
