# Supplementary material for: Prospective evaluation of Gadoxetate-enhanced magnetic resonance imaging and computed tomography for hepatocellular carcinoma detection and transplant eligibility assessment with explant histopathology correlation
Source: Cancer Imaging. 2023 Feb 25;23:22. doi: 10.1186/s40644-023-00532-3 (PMC9960413; doi:10.1186/s40644-023-00532-3)
Supplement: Supplementary file 1 — Additional file 1. Multiphasic Liver CT protocol (Aquilion 64). [file 40644_2023_532_MOESM1_ESM.docx]

**Supplementary Table 1 Multiphasic Liver CT protocol (Aquilion 64)**

| CT Scanner parameters |  |
| --- | --- |
| Axial slice thickness | 5 mm |
| Slice interval | 2.5 mm |
| Detector configuration | 64 × 0.5 (32 mm) |
| Tube voltage | 120 kV |
| Automated tube current | 80-440 mA |
| Helical pitch | 53 |
| Pitch factor | 0.828 |
| Tube rotation time | 0.5 s |
| Craniocaudal coverage | Unenhanced, arterial and delayed phases: from top of the diaphragm to bottom of liver  Venous phase: from top of the diaphragm to below symphysis pubis |
| IV Contrast^*^ | Ultravist 370, GE Healthcare, 2ml/kg to a maximum of 150 mL |
| Phase Delay | Phase 1: no delay  Phase 2: Arterial - Sure Start on abdominal aorta at 100 HU plus  25 sec delays  Phase 3: Venous - 100HU plus 60 sec delays  Phase 4: 180 sec delays |
| Multiplanar reformatted images | Coronal and sagittal planes for all phases with a slice thickness of 3 mm |

* Contrast was administered intravenously, through a minimum 20-gauge intravenous catheter inserted into a forearm vein, with a power injector [Medrad® Stellant® Dual Head] at a rate of 5mL/s.
