# Supplementary material for: Prospective evaluation of Gadoxetate-enhanced magnetic resonance imaging and computed tomography for hepatocellular carcinoma detection and transplant eligibility assessment with explant histopathology correlation
Source: Cancer Imaging. 2023 Feb 25;23:22. doi: 10.1186/s40644-023-00532-3 (PMC9960413; doi:10.1186/s40644-023-00532-3)
Supplement: Supplementary file 2 — Additional file 2. MRI Protocol for Gadoxetic acid-enhanced liver MRI (EOB-MRI). [file 40644_2023_532_MOESM2_ESM.docx]

**Supplementary Table 2 MRI Protocol for Gadoxetic acid-enhanced liver MRI (EOB-MRI)**

| **Image sequence** | **Field strengths** | **TR (ms)** | **TE (ms)** | **FA (D^◦^)** | **FOV (mm)** | **ST (mm)** | **Voxel size (mm)** | **Fat suppression** | **Respiratory control** | |
| --- | --- | --- | --- | --- | --- | --- | --- | --- | --- | --- |
| **Axial T2 HASTE** | **1.5T** | 1200 | 180 | 160 | 370 | 5 | 1.2 x 1.2 x 5.0 | SPAIR | Breath-hold | |
|  | **3T** | 1200 | 181 | 146 | 380 | 5 | 1.2 x 1.2 x 5.0 |  |  |  |
| **Coronal T2 HASTE** | **1.5T** | 1200 | 180 | 160 | 370 | 4 | 1.2 x 1.2 x 4.0 | None | Breath-hold | |
|  | **3T** | 1600 | 180 | 160 | 380 | 4 | 1.2 x 1.2 x 4.0 |  |  |  |
| **Axial T1 VIBE in-out phase** | **1.5T** | 150 | 1.45-2.69 | 70 | 370 | 4 | 0.7 x 0.7 x 4.0 | None | Breath-hold | |
|  | **3T** | 150 | 1.23-2.46 | 70 | 380 | 4 | 0.7 x 0.7 x 4.0 |  |  |  |
| **DWI ep2d diff b100,600** | **1.5T** | 6300 | 63.0 | - | 400 | 5 | 1.0 x1.0 x 5.0 | SPAIR | Off | |
|  | **3T** | 6600 | 68.0 | - | 400 | 5 | 1.0 x 1.0 x 5.0 |  |  |  |
| **Axial T1 VIBE** | **1.5T** | 4.10 | 1.98 | 10 | 370 | 3 | 1.2 x 1.2 x 3.0 | SPAIR | Breath-hold | |
|  | **3T** | 3.60 | 1.75 | 9 | 380 | 3 | 1.2 x 1.2 x 3.0 |  |  |  |
| **Post-contrast imaging*** | | | | | | | | | |  |
| **Axial T1 VIBE**  **(arterial phase)** | **1.5T** | 3.56 | 1.74 | 10 | 380 | 3 | 1.2 x 1.2 x 3.0 | SPAIR | Breath-hold | |
|  | **3T** | 3.60 | 1.75 | 9 | 380 | 3 | 1.2 x 1.2 x 3.0 |  |  |  |
| **Axial T1 VIBE**  **(portal venous phase)** | **1.5T** | 4.10 | 1.98 | 10 | 370 | 3 | 1.2 x 1.2 x 3.0 | SPAIR | Breath-hold | |
|  | **3T** | 3.60 | 1.75 | 9 | 380 | 3 | 1.2 x 1.2 x 3.0 |  |  |  |
| **Axial T1 VIBE**  **(transitional phase)** | **1.5T** | 4.10 | 1.97 | 10 | 380 | 3 | 1.2 x 1.2 x 3.0 | SPAIR | Breath-hold | |
|  | **3T** | 3.60 | 1.75 | 9 | 380 | 3 | 1.2 x 1.2 x 3.0 |  |  |  |
| **Axial T1 VIBE 20 min**  **(hepatobiliary phase)** | **1.5T** | 4.29 | 1.73 | 25 | 370 | 3 | 1.2 x 1.2 x 3.0 | Q-fat | Breath-hold | |
|  | **3T** | 4.29 | 1.82 | 25 | 360 | 3 | 1.1 x 1.1 x 3.0 |  |  |  |
| **Coronal T1 VIBE 20 min** | **1.5T** | 4.39 | 1.67 | 25 | 420 | 3 | 1.3 x 1.3 x 3.0 | Q-fat | Breath-hold | |
|  | **3T** | 4.39 | 1.75 | 25 | 420 | 3 | 1.3 x 1.3 x 3.0 |  |  |  |
| **Axial T1 CAIPI-VIBE 20 min (hepatobiliary phase)** | **1.5T** | 4.29 | 1.74 | 25 | 360 | 3 | 1.1 x 1.1 x 3.0 | Q-fat | Breath-hold | |
|  | **3T** | 4.02 | TE1 = 1.32  TE2 = 2.55 | 9 | 360 | 1.5 | 1.1 x 1.1 x 1.5 |  |  |  |

*Gadoxetic acid (Primovist or Eovist, Bayer AG, Germany) was administered to patients intravenously through a 22-gauge intravenous catheter inserted into a forearm vein with an MR-compatible power injector (Medrad® Spectris Solaris® EP MR Injection system, Bayer Healthcare, Whippany, USA) at a rate of 1 mL/s (0.025 mmol/kg body weight) followed by a 10-mL normal saline chaser at the same rate.

FA: flip angle, FOV: field of view, SPAIR: Spectral Attenuated Inversion Recovery, ST: section thickness, TE: echo time, TR: repetition time
