# Supplementary material for: Prospective evaluation of Gadoxetate-enhanced magnetic resonance imaging and computed tomography for hepatocellular carcinoma detection and transplant eligibility assessment with explant histopathology correlation
Source: Cancer Imaging. 2023 Feb 25;23:22. doi: 10.1186/s40644-023-00532-3 (PMC9960413; doi:10.1186/s40644-023-00532-3)
Supplement: Supplementary file 3 — Additional file 3. LI-RADS v2018 – Major and Ancillary Features with EOB-MRI. [file 40644_2023_532_MOESM3_ESM.docx]

**Supplementary Table 3 LI-RADS v2018 – Major and Ancillary Features with EOB-MRI**

| **Major features** | Non-rim arterial hyperenhancement, Washout appearance on PP, Enhancing capsule |
| --- | --- |
| **Ancillary features favoring malignancies in general** | Mild to moderate T2 hyperintensity, HBP hypointensity, Restricted diffusion, |
| **Ancillary features favoring HCCs in particular** | Nodule-in-nodule architecture, Fat in mass, Blood product in mass |
| **Imaging features favoring non-HCC malignancies** | Targetoid dynamic enhancement including AP rim enhancement, peripheral washout on PVP, delayed centripetal enhancement, targetoid TP or HBP appearance, and targetoid restriction |

AP: arterial phase, EOB-MRI: Gadoxetic acid-enhanced MRI, HBP: hepatobiliary phase, PVP: portal venous phase, TP: transitional phase
