# Supplementary material for: Prospective evaluation of Gadoxetate-enhanced magnetic resonance imaging and computed tomography for hepatocellular carcinoma detection and transplant eligibility assessment with explant histopathology correlation
Source: Cancer Imaging. 2023 Feb 25;23:22. doi: 10.1186/s40644-023-00532-3 (PMC9960413; doi:10.1186/s40644-023-00532-3)
Supplement: Supplementary file 4 — Additional file 4. Lesion characteristics evaluated on EOB-MRI. [file 40644_2023_532_MOESM4_ESM.docx]

**Supplementary Table 4 Lesion characteristics evaluated on EOB-MRI**

| Size (centimeter) |  |
| --- | --- |
| Location^*^ | Couinaud segmental anatomy |
| Reference image | Series/image |
| SI on precontrast T1W | Low, iso, high |
| Postcontrast T1W dynamic (AP, PP) | Low, iso, high |
| SI on T2W | Low to intermediate, high, very high |
| SI on HBP | Low, iso, high |
| Pattern of enhancement | Peripheral vs non-peripheral |
| Pattern of washout | Peripheral vs non-peripheral |
| Enhancing capsule | Yes/No |
| Non-enhancing capsule | Yes/No |
| Restricted diffusion | Yes/No |
| Intralesional fat | Yes/No |
| Intralesional iron | Yes/No |
| Intralesional blood products | Yes/No |
| Nodule-in-nodule | Yes/No |
| Mosaic architectures | Yes/No |

* Readers recorded references to image slices for each observation. AP: arterial phase, EOB-MRI: Gadoxetic acid-enhanced MRI, HBP: hepatobiliary phase, PVP: portal venous phase, SI: signal intensity, T1W: T1-weighted, T2W: T2-weighted
