# Supplementary material for: Prospective evaluation of Gadoxetate-enhanced magnetic resonance imaging and computed tomography for hepatocellular carcinoma detection and transplant eligibility assessment with explant histopathology correlation
Source: Cancer Imaging. 2023 Feb 25;23:22. doi: 10.1186/s40644-023-00532-3 (PMC9960413; doi:10.1186/s40644-023-00532-3)
Supplement: Supplementary file 5 — Additional file 5. Lesion characteristics evaluated on Contrast-enhanced CT scan. [file 40644_2023_532_MOESM5_ESM.docx]

**Supplementary Table 5 Lesion characteristics evaluated on Contrast-enhanced CT scan**

| Size (centimeter) |  |
| --- | --- |
| Location^*^ | Couinaud segmental anatomy |
| Reference image | Series/image |
| Precontrast density | Low, iso, high |
| Postcontrast density (AP, PVP, DP) | Low, iso, high |
| Pattern of enhancement | Peripheral vs non-peripheral |
| Pattern of washout | Peripheral vs non-peripheral |
| Enhancing capsule | Yes/No |
| Non-enhancing capsule | Yes/No |
| Intralesional fat | Yes/No |
| Intralesional blood products | Yes/No |
| Nodule-in-nodule | Yes/No |
| Mosaic architectures | Yes/No |

* Readers recorded references to image slices for each observation. AP: arterial phase, DP: delayed phase, PVP: portal venous phase
