# Supplementary material for: Prospective evaluation of Gadoxetate-enhanced magnetic resonance imaging and computed tomography for hepatocellular carcinoma detection and transplant eligibility assessment with explant histopathology correlation
Source: Cancer Imaging. 2023 Feb 25;23:22. doi: 10.1186/s40644-023-00532-3 (PMC9960413; doi:10.1186/s40644-023-00532-3)
Supplement: Supplementary file 6 — Additional file 6. CECT and EOB-MRI treatment response categories. [file 40644_2023_532_MOESM6_ESM.docx]

**Supplementary Table 6 CECT and EOB-MRI treatment response categories**

| Response Category | Criteria |
| --- | --- |
| LR-TR Viable | Nodular, masslike, or thick irregular tissue in or along the treated lesion with APHE or washout, or enhancement similar to pretreatment |
| LR-TR Equivocal | Enhancement pattern not typical for treatment specific expected enhancement and not meeting the criteria for a viable lesion |
| LR-TR Nonviable | No lesional enhancement or treatment specific expected enhancement pattern |
| LR-TR Nonevaluable | Not evaluable due to image omission or degradation |

CECT: contrast-enhanced CT scan, EOB-MRI: Gadoxetic acid-enhanced MRI
