# Supplementary material for: Prospective evaluation of Gadoxetate-enhanced magnetic resonance imaging and computed tomography for hepatocellular carcinoma detection and transplant eligibility assessment with explant histopathology correlation
Source: Cancer Imaging. 2023 Feb 25;23:22. doi: 10.1186/s40644-023-00532-3 (PMC9960413; doi:10.1186/s40644-023-00532-3)
Supplement: Supplementary file 7 — Additional file 7. OPTN classification system for lesions seen on imaging of cirrhotic livers. [file 40644_2023_532_MOESM7_ESM.docx]

**Supplementary Table 7 OPTN classification system for lesions seen on imaging of cirrhotic livers**

| 0 | Incomplete or technically inadequate study |
| --- | --- |
| 5A | Maximum diameter of ≥ 1 cm and < 2 cm, as measured on late arterial or portal phase images.  • Increased contrast enhancement relative to hepatic  parenchyma on the late arterial phase.  • Washout during the later contrast phases and peripheral  rim enhancement on delayed phase |
| 5B | Must meet all of the following:  1. Maximum diameter of ≥ 2 cm and ≤ 5 cm, as measured on late arterial or portal phase images.  2. Increased contrast enhancement relative to hepatic  parenchyma on the late arterial phase.  3. One of the following:  a. Washout on portal venous/delayed phase.  b. Peripheral rim enhancement. |
| 5T | Any Class 5A and 5B lesion that has subsequently been ablated. |

OPTN: Organ Procurement and Transplantation Network
