# Supplementary material for: Prospective evaluation of Gadoxetate-enhanced magnetic resonance imaging and computed tomography for hepatocellular carcinoma detection and transplant eligibility assessment with explant histopathology correlation
Source: Cancer Imaging. 2023 Feb 25;23:22. doi: 10.1186/s40644-023-00532-3 (PMC9960413; doi:10.1186/s40644-023-00532-3)
Supplement: Supplementary file 8 — Additional file 8. Distribution of non-treated hepatocellular carcinoma (HCC) and viable HCC in explants. [file 40644_2023_532_MOESM8_ESM.docx]

**Supplementary Table 8 Distribution of non-treated hepatocellular carcinoma (HCC) and viable HCC in explants**

| **Number of non-treated HCC in an explant** | **Number of patients** | **Number of viable HCCs in an explant** | **Number of patients** |
| --- | --- | --- | --- |
| 0 | 27 | 0 | 29 |
| 1 | 9 | 1 | 24 |
| 2 | 12 | 2 | 5 |
| 3 | 4 | 3 | 1 |
| 4 | 1 | 4 | 0 |
| 5 | 3 | 5 | 1 |
| >5 | 4^a^ | >5 | 0 |
| **Total** | 60 | **Total** | 60 |

HCCs: hepatocellular carcinomas

^a^ Two explants harbored 10 HCCs each, while the other two explants had seven and thirteen HCCs.
