# Supplementary material for: Prospective evaluation of Gadoxetate-enhanced magnetic resonance imaging and computed tomography for hepatocellular carcinoma detection and transplant eligibility assessment with explant histopathology correlation
Source: Cancer Imaging. 2023 Feb 25;23:22. doi: 10.1186/s40644-023-00532-3 (PMC9960413; doi:10.1186/s40644-023-00532-3)
Supplement: Supplementary file 9 — Additional file 9. Histopathology results for LR-2 and LR-3 lesions scored by readers on EOB-MRI and CECT. [file 40644_2023_532_MOESM9_ESM.docx]

**Supplementary Table 9 Histopathology results for LR-2 and LR-3 lesions scored by readers on EOB-MRI and CECT**

| **Pathology** | **R1-EOB-MRI** | | **R2-EOB-MRI** | | **R1-CECT** | | **R2-CECT** | |
| --- | --- | --- | --- | --- | --- | --- | --- | --- |
|  | **LR-2** | **LR-3** | **LR-2** | **LR-3** | **LR-2** | **LR-3** | **LR-2** | **LR-3** |
| **HCC** | 1 | 2 | 1 | 11 | 1 | 8 | 4 | 9 |
| **Benign** | 4 | 0 | 4 | 0 | 4 | 0 | 4 | 0 |
| **Total** | 5 | 2 | 5 | 11 | 5 | 8 | 8 | 9 |

CECT: contrast-enhanced CT scan, EOB-MRI: Gadoxetic acid-enhanced MRI, LR: LI-RADS, R1: reader 1, R2: reader 2
